# Nanomotion detection based on atomic force microscopy cantilevers

**DOI:** 10.1016/j.tcsw.2019.100021

**Published:** 2019-03-08

**Authors:** A.C. Kohler, L. Venturelli, G. Longo, G. Dietler, S. Kasas

**Affiliations:** a Laboratoire de Physique de la Matière Vivante, EPFL, CH-1015 Lausanne, Switzerland; b Istituto di Struttura della Materia ISM-CNR, Rome, Italy; c Unité Facultaire d’Anatomie et de Morphologie, CUMRL, Université de Lausanne, CH-1005 Lausanne, Switzerland

**Keywords:** AFM, Nanomotion detection, Cell viability, Antibiotic Susceptibility Test (AST)

## Abstract

Atomic force microscopes (AFM) or low-noise in-house dedicated devices can highlight nanomotion oscillations. The method consists of attaching the organism of interest onto a silicon-based sensor and following its nano-scale motion as a function of time. The nanometric scale oscillations exerted by biological specimens last as long the organism is viable and reflect the status of the microorganism metabolism upon exposure to different chemical or physical stimuli. During the last couple of years, the nanomotion pattern of several types of bacteria, yeasts and mammalian cells has been determined. This article reviews this technique in details, presents results obtained with dozens of different microorganisms and discusses the potential applications of nanomotion in fundamental research, medical microbiology and space exploration.

## 1 Introduction

The atomic force microscope (AFM) was invented in 1986 by Binnig et al. (1986). The instrument consists of a micro-fabricated tip attached at the end of a soft cantilever,. Using high-resolution piezo actuators, the tip is approached to the sample until the interaction forces between the atoms of the tip and those of the sample induce a deformation of the cantilever. The cantilever deformations are detected through the deflection of a laser beam reflected at the end of the cantilever, which then illuminates a two or four segments photodiode. This force sensor scans the surface of the sample while the deformations of the cantilever are recorded, and this raster scan reconstructs the 3D topography of the surface. This detection system is highly sensitive and sub-Angstrom cantilever deformations can be monitored (Scheuring et al., 1999)**.** The device operates indifferently in vacuum, air or liquid at various temperature (Drake et al., 1989). This last feature makes it particularly appealing for biological sample measurements since most of the biological reactions take place at 37 °C and in solution.

The range of application of the instrument are not limited to high-resolution imaging. It can also collect additional information about the stiffness of the sample. One of the most popular technique to conduct such measurement consist in indenting (i.e. pushing) the AFM tip (cantilever that can be functionalized with proteins or molecules) into the sample and monitoring the cantilever deflection as a function of the sample-penetration depth. This type of measurement is widely used nowadays to characterize the mechanical properties ranging from single molecules to whole mammalian cells (Krieg et al., 2018). For a more in depth explanation of this method, additional readings can be found in the following articles (Kasas et al., 2015a, Stupar et al., 2017a). Furthermore, the instrument can also assess the presence and properties of specific molecules on the scanned sample. The aforementioned application is accomplished by attaching the ligands of the molecules to be located onto the AFM tip and in monitoring attachment/detachment events during the scan (Haase and Pelling, 2015, Japaridze et al., 2017, Smolyakov et al., 2016). A recent and comprehensive review about the AFM applications in the field of microbiology can be found in (Formosa-Dague et al., 2018).

More recently, a new technique using AFM has emerged. It consists of attaching biological specimens directly onto the cantilever. In such case the added mass induces changes in the surface stress, the static bending and/or the resonance frequency of the cantilever (Boisen, 2011, Tamayo et al., 2013, Zangle and Teitell, 2014). This method allows to precisely monitor the attachment of single molecules to the cantilever and to measure the changes in mass of a single living cell (Braun et al., 2009, Ilic et al., 2000, Martínez-Martín et al., 2017, Ramos et al., 2008).

**Fig. 1:**
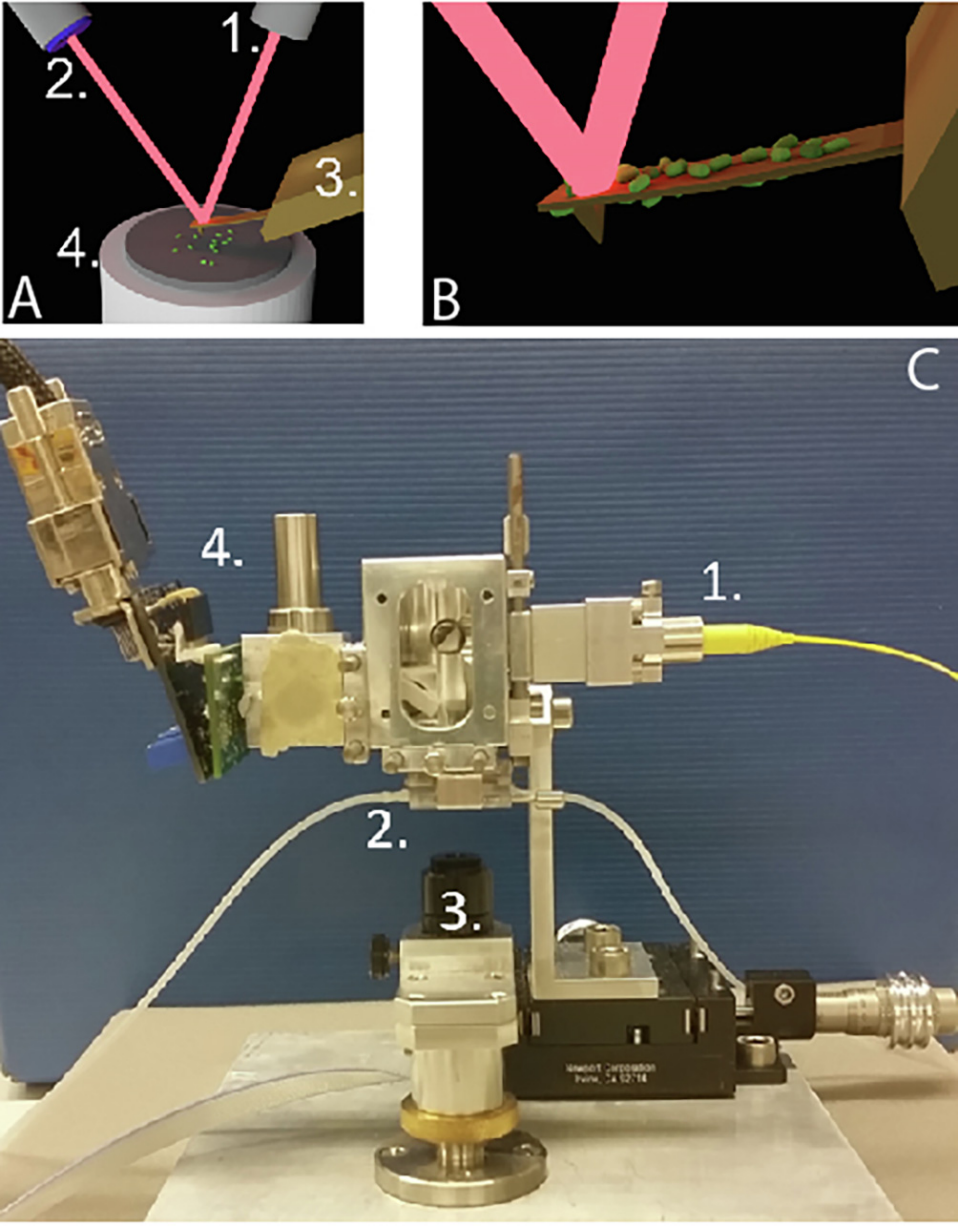
. Principle of AFM and nanomotion detection **A**: An AFM cantilever (3) is scanning a surface (4) with living organisms and their topographic characteristics are recorded by the means of the laser displacement (1) by a 4-quadrant photodiode (2). **B**: basic AFM-based nanomotion detection. The living organisms are attached onto the functionalized cantilever and its oscillations are followed as in a normal AFM procedure. **C**: Example of an in-house made nanomotion detector. 1: laser, 2: analysis chamber with AFM cantilever (not visible in this image), 3: optical microscope and CCD camera, 4: photodetector and preamplifier.

In 2013, with a similar technique Longo et al (2013) were able to detect nanomotion of living organism. The cells of interest were attached to the cantilever and immersed in a liquid filled analysis chamber. The deflection of the cantilever was then monitored upon exposure to different chemical susceptible to affect their metabolism or viability (Fig. 1A and B). This technique is referred to as AFM-based nanomotion detection. In this review, we will highlight the recent progress made regarding nanomotion detection and its potential application.

## 2 AFM-based nanomotion detection

The mechanism behind the detection of these oscillations relies on the mechanical properties of the cantilever and the highly sensitive detection method (described in the introduction). Depending on the spring constant of the cantilever, the sensitivity of the nanomotion detection system can varies. A cantilever with low spring constant will be more sensitive to bending and more prone to register environmental noise, compared to a cantilever with a higher spring constant. Typically, cantilevers dedicated for biological applications are well adapted for nanomotion detection. They have a spring constant of 0.01 to 0.5 N/m and a resonant frequency ranging from 3 to 30 kHz. Furthermore, the last parameter strongly depends on the viscosity of the media in which the cantilever is immersed. Cantilevers that do not have biological samples on their surface spontaneously oscillate due to thermal and Brownian motion. These oscillations have a lower amplitude compared to the oscillation that are detected once living samples have been attached to the cantilever (Longo et al., 2013a).

The very first example of AFM cantilevers used to detect molecular oscillations was carried out by Radmacher et al., in 1994. In this seminal work, the authors adsorbed lysozyme molecules onto a mica surface and moved the AFM tip above the protein layer. Upon exposure of lysozyme to one of its substrates (e.g oligoglycoside), the cantilever was set to oscillate (Radmacher et al., 1994). The authors speculated that the oscillations were induced by lysozyme conformational changes. Several years later, Alonso et al., coated an AFM cantilever with topoisomerase II, a protein that participates in the DNA unfolding (Salceda et al., 2006) by changing conformation by oxidizing ATP, and that is part of the mitotic chromosome scaffold (Farr et al., 2014). By recording the oscillations of the cantilever at different ATP concentrations, the authors were able to measure a drastic increase in the fluctuation of the amplitude of the deflection of the cantilever after an increase of the ATP concentration. This suggests that the oscillations were induced by the ATP-triggered Topoisomerase II conformational changes and by energy dissipation caused by the ATP hydrolysis (Alonso-Sarduy et al., 2014).

Nanoscale vibrations were also exploited to evaluate the viability of different microorganisms. The first report of this is by Pelling et al., 2004, who demonstrated how a constituent of living yeast cells (i.e the cell wall) produce a measurable vibration. In this study, an AFM tip was brought in close contact with the cell wall of *Saccharomyces cerevisiae*, which induced nanoscale oscillations of the cantilever at a relatively high frequency. Additional characterizations showed that the recorded signal was not due to Brownian motion or noise but had a biological origin (Pelling et al., 2004). Since then, several studies have explored the nanomotion of various microscopic organisms. In 2013 Longo et al., demonstrated that bacteria attached onto an AFM cantilever induce oscillations that are directly correlated to the organism viability. The time-dependent chart of the vertical movements of the sensor form a coloured noise signal, a nanomotion pattern, whose amplitude can provide a real-time determination of the metabolic status of the specimens as a function of different physico-chemical stimuli and can, therefore, be used to distinguish almost instantaneously life–death transitions or the real-time behavior of the microorganism. Remarkably the very same observations were reproduced by different groups on bacteria (Domínguez et al., 2015, Etayash et al., 2016, Lissandrello et al., 2014, Yang et al., 2017) and on other biological systems (Alonso-Sarduy et al., 2014, Kasas et al., 2015a, Wu et al., 2016, Wu et al., 2017), validating the nanomotion sensor in research laboratories and highlighting its robustness.

**Fig. 2:**
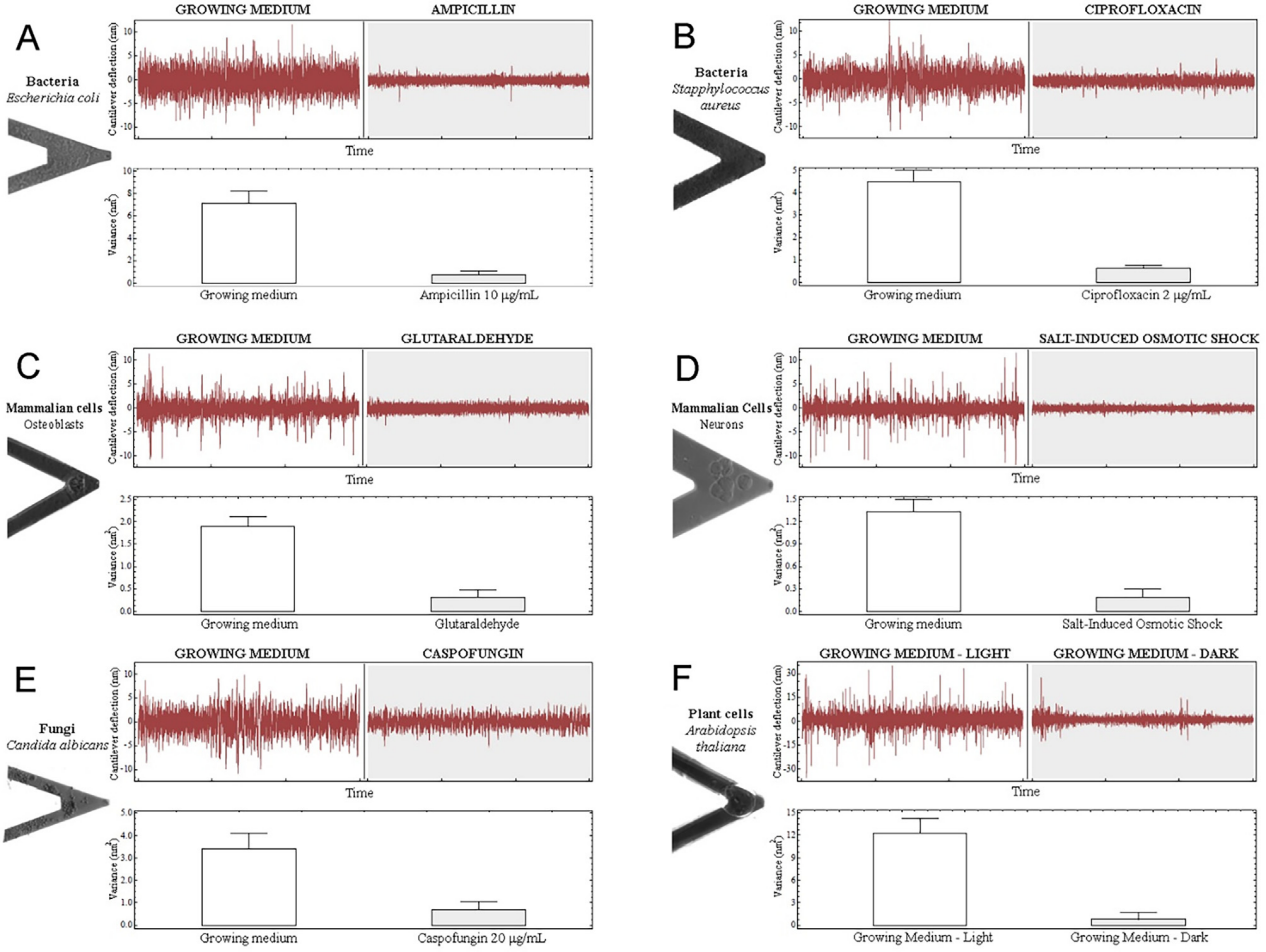
. Versatility of nanomotion to determine the viability of cells Nanomotion of several organisms were determined in different conditions. These graphs represent nanomotion of bacteria (A, B), fungi (E), mammalian (C, D) and plant cells (F) in growth media and after addition of molecules that alter their viability. In all the type of cells tested, the cantilever deflection as well as the variance of the cantilever deflection drastically decrease in presence of these specific molecules: antibiotics for bacteria (A, B), cross-linking agent or osmotic shock for C and D respectively, antifungal for E and exposure to darkness for plant cells. The picture of the cantilever highlights the presence of the cells throughout the experiment, providing evidence that the decrease is not due to loss of cells from the cantilever. Figure adapted from PNAS, January13,2015,112,2,378–391 with authorization.

The experimental procedure is relatively simple: the organism of interest is attached onto the cantilever by using molecules such as glutaraldehyde, polylysine, fibronectin, APTES or laminin. Importantly these agents must promote the adhesion of the organism without compromising its viability. The number of cells needed to detect oscillations is typically very low (Alonso-Sarduy et al., 2014) and depends on their size. A single yeast or mammalian cell is enough to obtain exploitable data whereas dozens to hundreds of bacteria are necessary to obtain equivalent signal (Kasas et al., 2015a). Once the living cells are attached to the cantilever, it is then immersed into the analysis chamber that contains growth medium. Finally, the oscillations of the cantilever are recorded before and after exposure of the sample to molecules that compromise the metabolism or the viability of the organism (Fig. 2). Typical experiments recorded on different type of cells are depicted in Fig. 2. All the organisms were exposed to chemicals compromising the survival of the cells: antibiotics and antifungal among others. As it can be noticed, the cantilever deflection of the signal drastically drops upon exposure to such drugs (Fig. 2).

### 2.1 Cellular mechanisms inducing nanomotion:

**Fig. 3:**
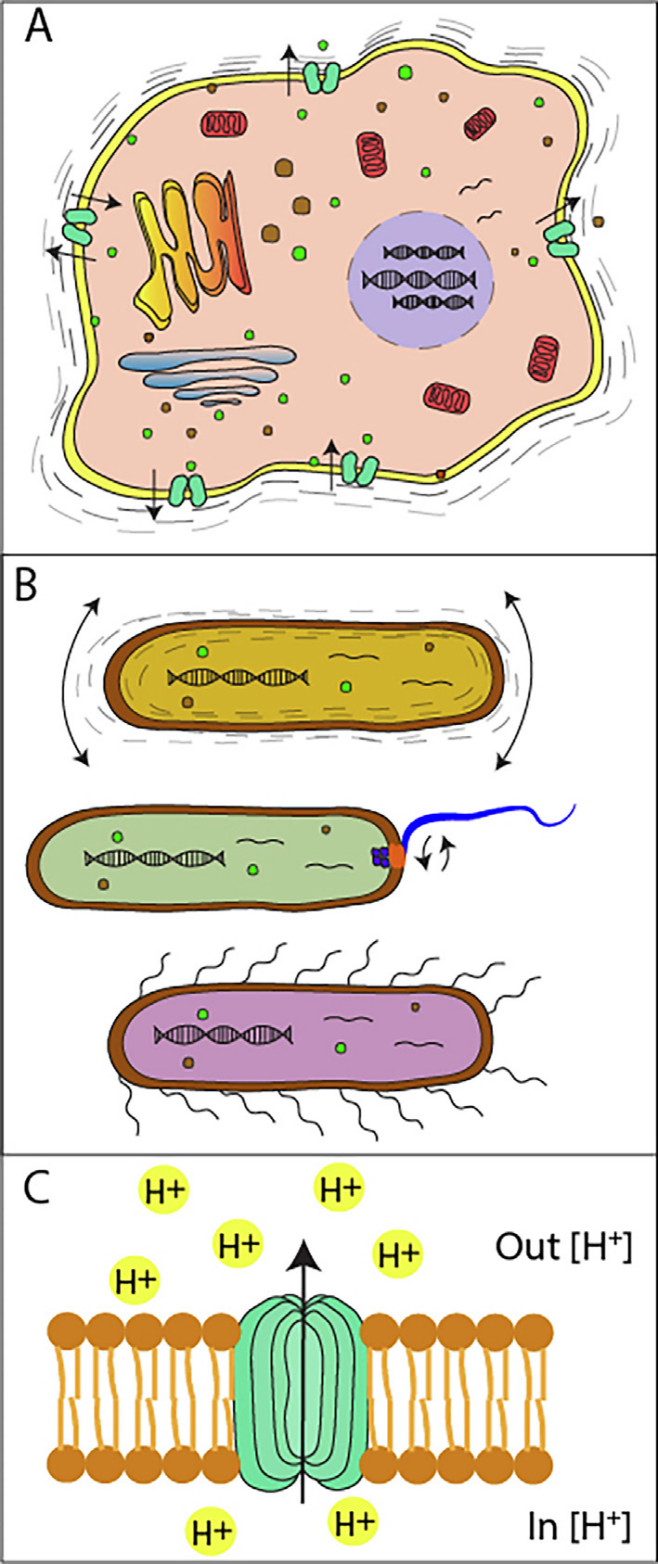
. Mechanism susceptible to cause cell nanomotion **A:** several molecular and cellular mechanism could be involved in the nanomotion of a cell. Among them, mitochondria and other metabolic processes could generate motion of the overall cell. Cell membrane motion itself could be part of the numerous mechanisms causing cellular nanomotion. **B:** extra-cellular organelles – such as pili or flagella – are motile components used for the locomotion of cells or bacteria this inherently to its function causes nanomotion of cells or bacteria. **C:** ion channels (green) change conformation in order to let in or out specific ions (depicted as H + in yellow). This structure rearrangement affects the lipid bilayer of cells and could be part of the nanomotion of cells. (For interpretation of the references to colour in this figure legend, the reader is referred to the web version of this article.)

Despite the aforementioned studies demonstrating that cantilever deflection is induced by nanomotion of living organism, its origin remains not fully understood. Several hypothesis exist to explain the phenomenon. The nanomotion signal is made of vibrations arising from many metabolically-related sources that combine energy consumption with local movement or molecule redistribution. These include protein cytoskeleton reorganization, focal adhesion movements, metabolically active organelles (such as mitochondria in animals and chloroplast in plants), vesicles production and trafficking, gating of ion channels, membrane interaction with the sensor or conformational changes of individual proteins (Fig. 3) (Alonso-Sarduy et al., 2014). All these biological signals sum up to produce nanomechanical oscillations and highlight the complexity of understanding the molecular and cellular mechanism behind nanomotion of cells.

A first hypothesis to explain cellular nanomotion involves the most external cellular component: the cell membrane for mammalian cells or cell wall for plant cells and microorganisms. Cell membranes are highly dynamic entities composed of a lipid bilayer which role is to give flexibility of the membrane and proteins (Fig. 3A). For several years now, cell membrane has been showed to be a dynamic entity (McNiven and Ridley, 2006). Even early stages of cytotoxicity cause a change in the viscosity of the cell membrane and morphology, both affecting their adhesion to the cantilever and the ability of the membrane to transduce the innermost vibrations (Yang et al., 2017). Furthermore, in the case of mammalian cells, actin depolymerization drugs affect some components of the nanomotion signal demonstrating a correlation between the actin reorganization and the nanomotion signal. Recently, it has been confirmed by Long-Range Surface Plasmon Resonance that cell membrane oscillates within a range of nanometers (Yang et al., 2015). This finding suggests a potential link between the cantilever deflection and cell membrane motion. Indeed, it has been demonstrated that actin depolymerization drugs negatively affect cantilever oscillations, suggesting a correlation between the actin reorganization and the nanomotion signal (Kasas et al., 2015a).

**Fig. 4:**
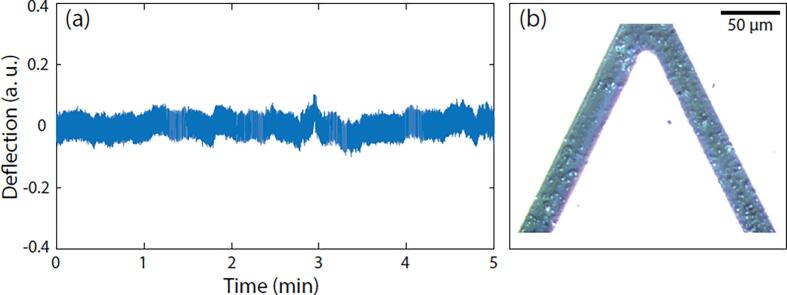
. Nanomotion signal induced by active mitochondria. Oscillations of the AFM cantilever as a function of time (a) with active mitochondria attached onto its surface (b). Reprinted from Stupar et al. (2017) Mech. Sci., 8, 23–28, 2017.

Intracellular organelles such as mitochondria and chloroplasts are involved in energy generation in cells. Preliminary experiments demonstrated that active mitochondria also induce cantilever oscillations and the oscillations generated by mitochondria change upon exposure to different molecules such as malate or pyruvate (Stupar et al., 2017a) (Fig. 4). Considering that mammalian cells possess multiple similar organelles in their cytoplasm, their individual activity could sum up and contribute to the resulting nanomotion signal (Fig. 3A).

Other factors that could affect nanomotion include motility-dedicated organelles. Some prokaryotes and eukaryotes possess flagella, pili or cilia (Fig. 3B). These structures are motile organelles that allow cells to move or to displace the liquid medium (such as the cilia in the respiratory tract epithelium). Their movement could transfer a momentum to the cantilever either directly or via liquid turbulences. For instance, it has been demonstrated that the inhibition of the movement of *E. coli* flagella results in a reduction of the nanomotion signal. It strongly indicates that some component of the signal are induced by these structures (Kasas et al., 2015a).

Another potential explanation for the origin of nanomotion is ion channels activities (Fig. 2C). Many organisms, including bacteria, yeast and plant cells possess thick and rigid cell walls that are less motile than the cell membrane of mammalian cells. Ion channels are proteins located in cell walls as well as in cell membranes and organelles (mitochondria and chloroplast) (Hille, 1978) which primary function is to maintain a concentration gradient of specific ions between the surrounding environment and the cytoplasm. Opening and closing an ion channel requires conformational changes (Sukharev et al., 2001) that can induce oscillations of the cantilever (Alonso-Sarduy et al., 2014).

This evidence of particular nanomotion features associated to particular metabolic cycles, has not yet translated to a direct indication of any specific frequency that is characteristic for a given organism or cellular component. The FFT spectrum of a typical nanomotion signal appears uniform and the biologically-related components are concentrated below 1 kHz (Lissandrello et al., 2014). This observation is valid for mammalian cells, yeasts and all the microorganisms explored until now with this technique.

## 3 Potential applications of the AFM based nanomotion detection

The first and most straightforward application of the technique is the rapid Antibiotic Sensitivity Test (AST). In the conventional hospital microbiological workflow, the clinical sample (*e.g.* blood, spinal fluid, urine, faeces, nasal or throat swab) is taken from the patient and streaked on agar nutrient media in the presence of the antibiotics to be tested. A 12–24-h long incubation period is required to obtain reproducible results. For slow growing bacteria such as *Bordetella pertussis* or *Mycobacterium tuberculosis*, the incubation time surpass the week (*e.g.* in the MGIT 960 system) or even the month (Horvat, 2010).

This long timeframe, under the pressure of life-threatening infections, often results in the use of broad-spectrum antibiotics.

A rapid AST could dramatically reduce the delay between the admission of the patient in a medical center and the administration of the appropriate treatment. Faster AST would not only increase the recovery rate of patients suffering from bacterial infection but also prevent the development of resistant strains. Applying nanomotion based AST in medical centers requires a high level of confidence in the technique. In a recent publication, Stupar et al. determined, within few hours, and in double blind experiments, the susceptibility of different bacterial strains to antibiotics with a success rate exceeding 94% (Stupar et al., 2017b). In addition to the sensitive/resistant response, the technique gives microbiologically relevant parameters such as MIC (minimum inhibitory concentration) and MBC (minimum bactericidal concentration) and provides information on the metabolic mechanisms that bacteria employ to react to an external agent. This is of utmost importance in the study of slowly growing bacteria, such as mycobacteria, where the determination of the MIC can require several months. Very recently Mustazzolu et al. have evaluated in few hours the response of a tuberculotic and a non-tuberculotic mycobacterial species to the exposure to specific antibiotics, determining the MIC and MBC. Furthermore, they exploited the high time resolution of the NMS to monitor the effect of the different drugs, evidencing the peculiar response of each bacterial species (Mustazzolu et al., 2019).

A second application of the technique is the rapid characterization of cancer cell sensitivity to antimitotic drugs. The test methodology consists in attaching cancer cells (1–5 of them) to the cantilever and to expose them to different antimitotic molecules. The most effective drugs and drug concentrations are selected according to their efficiency to compromise the viability of the cells. This approach could result in a more efficient way to treat patients, in the frame of a personalized medical treatment. The approach does not require additional cell culture step that often results in phenotypic changes of the cells and makes the response to drugs less reliable. The approach was first proposed by Kasas et al. (2015b) and further by Wu et al., demonstrated the efficiency of this technique. In this later work, the authors studied the effect of Paclitaxel (a potent antimitotic drug) on breast cancer cells and determined the minimum concentration needed to induce cell death (Wu et al., 2016).

Indeed, the fact that the nanomotion detection method could identify, in less than a few hours, the sensitivity of completely different specimens, such as bacteria or cancer cells, to a specific drug opens to interesting diagnostic applications. Besides, the nanomotion sensor allows fast and parallel preliminary screenings of new molecules to determine their effect on bacteria or eukaryotic cells (Imperi et al., 2014, Rampioni et al., 2017).

Other applications of the nanomotion sensor include characterization of organisms dwelling in extreme environments or in remote celestial bodies. A preliminary study has been performed by Kasas et al., to assess the presence of living organisms (Kasas et al., 2015a) in water and dry soil samples. One should keep in mind that metabolites as well the metabolic pathways used by extremophiles or extra-terrestrial organisms potentially differ from those encountered on Earth in normal condition. Therefore, an apparatus that detects living organisms in a chemistry independent manner would be a valuable tool to include in the instrumentation chain of remote-controlled devices exploring oceanic abysses or other planets.

As previously mentioned in this review, mitochondria nanomotion was assessed in the presence of different molecules altering their metabolism. Taken advantages of this setup, a diagnostic tool could be developed to detected mitochondria-linked disorders. Mitochondrial dysfunctions are difficult, time consuming and expensive to detect. Nanomotion based detection could offer a faster alternative as a diagnostic tool not only for mitochondria specific disorders but also for other metabolic related disorders.

Finally, Ruggeri et al., have applied this nanomotion detector to study the specific responses of neurons exposed to physiological concentrations of extracellular monomeric and toxic amyloid aggregated species of α-synuclein (Ruggeri et al., 2017) and work is underway to evaluate the effect of oxidative stress on different kinds of cells (Lupoli et al., 2018).

Other setups that do not involve an AFM have been recently developed for the exploitation of nanomotion sensors. They are based on optical and plasmonic resonance nanomotion (Syal et al., 2016, Syal et al., 2017) and require immuno-binding of the microorganisms on the sensing surfaces. While these setups were shown to be able to detect live and dead cell transitions, no clinical testing has yet been performed using these two alternative nanomotion systems.

## 4 Conclusion and future perspective

AFM based nanomotion detection is a new technique with interesting potential in different biomedical applications. Its speed and sensitivity make this system a unique tool to study living biological systems and their interactions with drugs. It has many clinical and research related applications, ranging from the determination of antibiotic treatments of bacterial infections, to the evaluation of the most appropriate anticancer drugs. Overall, its features make this an ideal innovative research platform to test new pharmacological methods and for drug development purposes as well as to study the response of cells to innovative chemical or physical stimuli.

Nanomotion based AST could significantly reduce the use of large spectrum antibiotics, shortening the treatment and rendering it more efficient. It will also help preventing and fighting the proliferation of antibiotic resistant bacteria. These expected results could be similar in terms of cancer drug susceptibility; despite the fact that data available in this field are still limited. The cellular mechanisms driving cellular nanomotion are not yet fully understood and further experiments will need to be performed to unveil the physiological mechanism that are fueling it. The increasing interest of this technique and its potential clinical applications makes it an exciting research domain to develop further and understand better AFM-based nanomotion detection.

## Conflict of interest

None.
